# Supplementary material for: A Critical View of the Application of the APEX Software (Aqueous Photochemistry of Environmentally-Occurring Xenobiotics) to Predict Photoreaction Kinetics in Surface Freshwaters
Source: Molecules. 2019 Dec 18;25(1):9. doi: 10.3390/molecules25010009 (PMC7017383; doi:10.3390/molecules25010009)
Supplement: Supplementary file 1 [file molecules-25-00009-s001.zip › 3.2.4_gcc-4.4.0.zip!/3.2.4_gcc-4.4.0/3.2.4_gcc-4.4.0/doc/octave/html/interpreter/Function-Handles.html]

Function Handles - Untitled


Next: Anonymous Functions,
Up: Function Handles Inline Functions and Anonymous Functions


---

#### 11.9.1 Function Handles

A function handle is a pointer to another function and is defined with
the syntax

```
     @function-name
```

For example

```
     f = @sin;
```

Creates a function handle called `f` that refers to the
function `sin`.

Function handles are used to call other functions indirectly, or to pass
a function as an argument to another function like `quad` or
`fsolve`. For example

```
     f = @sin;
     quad (f, 0, pi)
         => 2
```

You may use `feval` to call a function using function handle, or
simply write the name of the function handle followed by an argument
list. If there are no arguments, you must use an empty argument list
(). For example

```
     f = @sin;
     feval (f, pi/4)
         => 0.70711
     f (pi/4)
         => 0.70711
```

— Built-in Function: **functions** (fcn\_handle)  
> Return a struct containing information about the function handle
> fcn\_handle.

— Built-in Function: **func2str** (fcn\_handle)  
> Return a string containing the name of the function referenced by
> the function handle fcn\_handle.

— Built-in Function: **str2func** (fcn\_name)  
> Return a function handle constructed from the string fcn\_name.
